# Pirfenidone; can it be a new horizon for the treatment of pulmonary fibrosis in mustard gas-intoxicated patients?

**DOI:** 10.1186/2008-2231-21-13

**Published:** 2013-02-18

**Authors:** Nasim Zamani

**Affiliations:** 1 Department of Clinical Toxicology, Loghman Hakim Hospital, Shahid Beheshti University of Medical Sciences, Karegar Street, Tehran, Iran

**Keywords:** Pirfenidone, Mustard gas, Pulmonary fibrosis

## Abstract

Sulfur mustard is an alkylating substance still regarded as a threat in chemical warfare and terrorism. Lung parenchymal damage occurs in the most severe inhalational exposures. It accompanies an increased risk of respiratory tract carcinomas and chronic respiratory sequelae including chronic bronchitis, bronchiectasis, pulmonary fibrosis, interstitial lung disease, emphysema, and bronchiolitis obliterans. Pirfenidone is an antifibrotic with anti-inflammatory and anti hydroxyl radical activities which stabilizes pulmonary function in idiopathic pulmonary fibrosis patients. It can be suggested in chronically exposed soldiers or workers with signs and symptoms of pulmonary fibrosis to improve their quality of life and even prognosis.

## Introduction

Sulfur mustard (SM) is an alkylating substance still regarded as a significant threat in chemical warfare and terrorism. The organs that are most commonly affected by mustard are the eyes, skin, and respiratory tract. Even decades after exposure, severe long-term effects such as chronic obstructive lung disease, lung fibrosis, eye problems, abnormal pigmentation of the skin, and various forms of cancer have been diagnosed in these individuals. Mustard undergoes intramolecular cyclization to form a highly reactive sulfonium ion that alkylates sulfhydryl (−SH) and amino (−NH_2_) groups. Hoarseness, cough, sore throat, and chest pressure are common initial complaints, but lung parenchymal damage only occurs in the most severe inhalational exposures. Factory workers chronically exposed to mustard as well as soldiers exposed to it during chemical wars have been reported to have an increased risk of respiratory tract carcinomas as well as chronic respiratory sequelae including chronic bronchitis, bronchiectasis, pulmonary fibrosis, interstitial lung disease, emphysema, and bronchiolitis obliterans [1].

During world war I, airway injury had been reported in 75% of those exposed to it [1]. Many Iranian soldiers (about 40000 to 50000 during the war between Iran and Iraq in 1980–88) are also of those currently injured and have chronic respiratory diseases due to mustard gas [2]. In different studies, the late onset of lung injuries among Iranian soldiers with wartime exposure to SM has been reported to be between 42.5% and 95% [1,2]. In a review performed by Mansour Razavi and colleagues, the prevalence of chronic pulmonary complications has been mentioned to be between 45.8% to 100% in civilian populations, 42.5% to 95% in veterans, and almost 100% in the children [3]. The late complications of SM in 600 patients evaluated 19 years after exposure has been reported 80.7% in another study [4]. In a review done by Poursaleh and associates, respiratory problems have been mentioned to be the greatest cause of long-term disability among the people with combat exposure to SM [5]. In this study, honeycomb lung pathology as fibrosis and slightly increased tendency for development of lung cancer with a latency of 20 years have been mentioned as two potential chronic complications of SM.

Mild, severe, and focal fibrosis were present in 4.8%, 1.6%, and 4.8% of the patients [6]. The most common alleged mechanism of such injuries is damaging the epithelial layer of the lung and airways with subsequent release of inflammatory mediators [7].

 On the other hand, pirfenidone (5-methyl-1-phenyl H-pyridin-2-one) is a newly discovered antifibrotic with anti-inflammatory and anti hydroxyl radical activities [8]. Its probable mechanism of act is inhibition of transforming Growth Factor Beta production. Its anti-inflammatory effect is probably due to the amelioration of the effect of Tumor Necrosis Factor Alpha. It has been shown that pirfenidone stabilizes pulmonary function in idiopathic pulmonary fibrosis patients [8].

 It can be suggested that in the sulfur mustard-exposed soldiers or the workers who are chronically exposed to this hazardous material and show signs and symptoms of pulmonary fibrosis, administration of this medication can improve their quality of life and even prognosis. This approach, however, should be reserved for those with confirmed pulmonary fibrosis and not as a routine approach for all who have confronted SM. Besides, prospective case–control studies in this regard are warranted. Thank you.

## Competing interests

The author declares that he has no competing interests.
